# Assessment of the effect on blood loss and transfusion requirements when adding a polyethylene glycol sealant to the anastomotic closure of aortic procedures: a case–control analysis of 102 patients undergoing Bentall procedures

**DOI:** 10.1186/1749-8090-7-105

**Published:** 2012-10-08

**Authors:** Ehsan Natour, Michael Suedkamp, Otto E Dapunt

**Affiliations:** 1 Department of Cardiothoracic Surgery, University Medical Centre Groningen, Groningen, The Netherlands; 2 Department of Cardiothoracic Surgery, Universitaetsklinikum Freiburg, Freiburg, Germany; 3 Department of Cardiothoracic Surgery, Klinikum Oldenburg, Oldenburg, Germany

**Keywords:** Aortic repair, Blood loss, Transfusion, Economics, Surgical sealant

## Abstract

**Background:**

The use of CoSeal®, a polyethylene glycol sealant, in cardiac and vascular surgery for prevention of anastomotic bleeding has been subject to prior investigations. We analysed our perioperative data to determine the clinical benefit of using polyethylene glycol sealant to inhibit suture line bleeding in aortic surgery.

**Methods:**

From January 2004 to June 2006, 124 patients underwent aortic surgical procedures such as full root replacements, reconstruction and/or replacement of ascending aorta and aortic arch procedures. A Bentall procedure was employed in 102 of these patients. In 48 of these, a polyethylene glycol sealant was added to the anastomotic closure of the aortic procedure (sealant group) and the other 54 patients did not have this additive treatment to the suture line (control group).

**Results:**

There were no significant between-group differences in the demographic characteristics of the patients undergoing Bentall procedures. Mean EuroSCORES (European System for Cardiac Operative Risk Evaluation) were 13.7 ± 7.7 (sealant group) and 14.4 ± 6.2 (control group), p = NS. The polyethylene glycol sealant group had reduced intraoperative and postoperative transfusion requirements (red blood cells: 761 ± 863 *versus* 1248 ± 1206 ml, p = 0.02; fresh frozen plasma: 413 ± 532 *versus* 779 ± 834 ml, p = 0.009); and less postoperative drainage loss (985 ± 972 *versus* 1709 ± 1302 ml, p = 0.002). A trend towards a lower rate of rethoracotomy was observed in the sealant group (1/48 *versus* 6/54, p = 0.07) and there was significantly less time spent in the intensive care unit or hospital (both p = 0.03). Based on hypothesis-generating calculations, the resulting economic benefit conferred by shorter intensive care unit and hospital stays, reduced transfusion requirements and a potentially lower rethoracotomy rate is estimated at €1,943 per patient in this data analysis.

**Conclusions:**

The use of this polymeric surgical sealant demonstrated improved intraoperative and postoperative management of anastomotic bleeding in Bentall procedures, leading to reduced postoperative drainage loss, less transfusion requirements, and a trend towards a lower rate of rethoracotomy. Hypothesis-generating calculations indicate that the use of this sealant translates to cost savings. Further studies are warranted to investigate the clinical and economic benefits of CoSeal in a prospective manner.

## Background

Aortic repair and reconstruction (including aortic arch surgery and combined procedures), despite being major cardiac surgical procedures, are now commonplace. Worldwide, tens of thousands of procedures are performed each year for the treatment of aortic or thoracic aneurysms, occlusions or dissections [1].

One of the main complications with these types of surgical procedures is the intraoperative and postoperative bleeding at the anastomotic suture line. The likelihood of bleeding can be influenced by a range of factors including comorbidities, surgical history, anticoagulation therapies, the type of surgical procedure employed and individual patient risk. This type of bleeding complication presents a major challenge to intraoperative and postoperative haemostasis, and failure to achieve adequate haemostasis can lead to a longer operative time, a greater need for blood transfusion products and a higher risk of postoperative complications and rethoracotomy. These factors and complications are obviously detrimental to the patient and incur significant additional costs. The level of additional costs is dependent upon the need for rethoracotomies, length of stay in the intensive care unit (ICU), overall duration of hospital stay, and the additional use of medical equipment and medical services used to diagnose and resolve the complication. Considering the large number of procedures performed each year, the maintenance of intraoperative and postoperative haemostasis is essential not only for improving patient outcomes, but also for the reduction of these societal and healthcare costs.

Increasingly, surgical sealants such as polymeric polyethylene glycol (PEG) are being utilised in cardiac and vascular surgery to control anastamotic bleeding from high-pressure suture lines [2-4]. In several clinical and pre-clinical studies, PEG has been shown to provide rapid and strong sealing while maintaining flexibility and elasticity, and avoiding any disturbance of the wound closure [5,6]. This PEG is a fully synthetic sealant containing no human/animal proteins or gluteraldehyde and it does not induce any adverse tissue responses, exhibits minimal or no toxicity, and resorbs fully within four weeks [3,5].

The objective of this retrospective patient case–control analysis was to assess the effect on blood loss, transfusion requirements, and associated cost savings when adding a PEG sealant to the anastomotic closure of aortic procedures performed using a Bentall procedure.

## Methods

### Patients and study setting

Between January 2004 and June 2006, 124 consecutive patients underwent aortic-related surgical procedures including full root replacements, reconstruction and/or replacement of ascending aorta and aortic arch procedures in the Cardiothoracic Surgery Department at the Klinikum Oldenburg, Oldenburg, Germany. Patients were operated on by any one of six cardiothoracic surgeons using standardised procedures, three of whom routinely added a PEG sealant to the anastomotic closure of aortic procedures with the intent to enhance sealing and reduce blood loss. Of the 124 patients, 102 underwent Bentall procedures and therefore comprised a comparable cohort without procedures that may facilitate bleeding and thus influence between-group comparisons (e.g. deep hypothermic circulatory arrest). These patients were retrospectively divided into two study groups depending on the haemostatic procedure used by the attending surgeon:

**The sealant group** i.e. those that received PEG as a surgical sealant in addition to the suture line (standard procedure plus PEG sealant performed by three of the six surgeons). **The control group** i.e. those who did not receive any additional treatment to the suture line (standard procedure without the addition of a PEG sealant which was performed by the other three of the six surgeons).

## Materials

The PEG sealant used in this study was CoSeal® (Baxter Healthcare Corporation, Deerfield, IL, USA), which is composed of two synthetic PEGs, a dilute hydrogen chloride solution and a sodium phosphate/sodium carbonate solution. It is indicated for use in vascular reconstructions to achieve adjunctive haemostasis by mechanically sealing areas of leakage. At the time of administration, the mixed PEGs and solutions, which must be used within two hours of reconstitution, generate a biocompatible, strongly adherent hydrogel that forms a cohesive matrix on the tissue within 60 seconds, and fully resorbs over the subsequent weeks [7].

For patients in the sealant group, the PEG sealant was sprayed in a prophylactic way on clamped vascular prostheses in a thin homogeneous layer over the anastomotic sites; minimal volumes (2–4 ml) were applied to blotted or air-dried surfaces and allowed to stand for at least one minute before unclamping. Application was in accordance with the product instructions for use via a product-specific gas-driven spray device (CoSeal Easy Spray®, Air Enhanced Applicator, Baxter, Deerfield, IL, USA) [7]. Additional movie files 1 and 2 show the intraoperative application of PEG sealant in more detail (see additional file 1 and 2). CoSeal was the only product used for aortic suture-line sealing during the procedure (treatment group).

### Operative procedure

Routine preoperative and intraoperative care protocols were followed, and all patients were assessed prior to surgery to ensure that patients had a normal haemoglobin level and no hereditary coagulopathies. In elective surgeries, preoperative aspirin was discontinued one week prior to surgery. Patients only received blood substitution immediately prior to surgery in emergency circumstances. A drop in blood haemoglobin below the normal levels (men: 13.5–17.5 g/dL; women: 12.0–16.0 g/dL) was the trigger for intraoperative transfusion. Aortic repair and reconstruction was carried out using a Bentall procedure utilising a Medtronic Freestyle® stentless root prosthesis (Medtronic Inc. Minneapolis, MN). In summary, once the diseased aortic tissue and valve had been removed, proximal anastomosis was performed using 20–25 single interrupted 4/0 Ethibond® sutures in a single plane. Mobilised coronary buttons were then sewn end-to-side to the corresponding aortic sinus using a continuous 5/0 polypropylene suture. Finally, the cranial end of the bioprosthesis was sewn end-to-end to the aorta with a continuous 4/0 polypropylene suture, completing the root replacement. Due to the short root prosthesis of the Medtronic Freestyle® device used for this procedure, some patients required additional length, which was provided by inserting a vascular tube graft (Vascutek®, Renfrewshire, Scotland). Any further surgery was carried out according to standard procedures; deaeration was performed following clamp removal and protamine was administered upon cessation of bypass.

### Outcomes

The evaluation of the effect of added PEG sealant was based on the retrospective analysis of three key outcome measures:

1) requirement for transfusion based on volumes (ml) of red blood cells (RBC) and fresh frozen plasma (FFP) within the first 48 hours following surgery;

2) postoperative drainage volume (ml) within the first 48 hours following surgery; and

3) rate of rethoracotomy.

The same key measures were used to perform a hypothesis-generating calculation of the costs associated with the Bentall procedures employed, either with or without PEG sealant. Indirect economic benefit was retrospectively estimated, with respect to reduced transfusion requirements and rethoracotomy rate, based on the following typical costs at the Klinikum Oldenburg from January 2004 to June 2006: RBC, €200 per unit; FFP, €160 per unit; PEG sealant, €237 per 2 ml application; ICU stay, €400 per day; hospital stay, €150 per day; and rethoracotomy, €2000 per procedure (operative costs only).

### Statistical analysis

Clinical assessment data were presented as mean ± standard deviation (SD) and population characteristics as a percentage of each subgroup (treatment or control). The following statistical tests were used to compare treatment groups: Student’s t-tests (gender, age, weight, European System for Cardiac Operative Risk Evaluation [EuroSCORE], cardiopulmonary bypass [CPB] time, aortic cross clamp time, total operative duration [or time], drainage volume, duration of ICU and total hospital stay), Wilcoxon-Mann-Whitney tests (urgency of operation), chi-square tests (surgical history and Bentall procedure), or a two-proportion z-test (postoperative rethoracotomy). P values of <0.05 were considered to be significant.

## Results

Between January 2004 and June 2006, a total of 124 consecutive patients underwent aortic-related surgical procedures at the Department of Cardiothoracic Surgery, Klinikum Oldenburg, Oldenburg, Germany. Of these, 102 patients underwent Bentall procedures and were included in the analysis: 48 (47.1%) received PEG as a surgical sealant in addition to the suture line (sealant group) and 54 (52.9%) did not receive this additive treatment to the suture line (control group). Demographic profiles of the two patient groups were similar and showed no significant differences between the sealant and control groups (Table 1).

**Table 1:** **Demographic and perioperative characteristicsof the study population**

**Bentall procedure**	**PEG sealant (N =** **48)**	**Control (no sealant) (N** **= 54)**	**p-value**
**Demographic characteristics**			
Gender (male)	31	37	NS
Age (years)	66.9 ± 9.5	70.3 ± 10.9	NS
Weight (kg)	82.6 ± 15.1	78.9 ± 13.9	NS
Surgical history (n,%)	6 (12.5)	6 (11.1)	NS
Aortic-valve replacement	3	2	NS
CABG	3	4	NS
EuroSCORE	13.7 ± 7.7	14.4 ± 6.2	NS
**Preoperative characteristics**			
Urgency of surgery (%)			
Elective	42 (87.5)	46 (85.2)	NS
Urgent	4 (8.3)	6 (11.1)	NS
Emergent	2 (4.2)	2 (3.7)	NS
**Intraoperative characteristics**			
CPB time (min)	167.71 ± 64.24	155.94 ± 46.35	NS
Aortic clamp time (min)	109.71 ± 36.88	102.64 ± 26.52	NS
Total operative duration (min)	215.40 ± 69.35	231.78 ± 65.25	NS
**Postoperative characteristics**			
Drainage volume (ml, within 48 hours)	985 ± 972	1709 ± 1302	0.002
	(220–6500)	(325–7500)	
RBC (average per patient in ml)	761 ± 863	1248 ± 1206	0.02
FFP (average per patient in ml)	413 ± 532	779 ± 834	0.009
Rethoracotomy (n,%)	1 (2)	6 (11.1)	0.07
Due to diffuse bleeding	1	3	
Due to hematoma	0	3	
Duration of ICU stay (days)	4.2 ± 3.6	6.5 ± 6.3	0.03
Duration of total hospital stay (days)	16.2 ± 8.8	21.0 ± 12.2	0.03

PEG, polyethylene glycol; CABG, Coronary artery bypass graft; CPB, cardiopulmonary bypass; EuroSCORE, European System for Cardiac Operative Risk Evaluation; SD, standard deviation.

P-values calculated using the following statistical tests: Student’s *t*-test, chi-square test, Wilcoxon-Mann-Whitney test, or Fisher’s exact test. All values are presented as mean ± SD or percentages of subgroup populations.

Compared with the control group, patients in the sealant group required significantly fewer intraoperative and postoperative transfusions of RBC (mean 761 ± 863 *versus* 1248 ± 1206 ml, p = 0.02; Table 1) or FFP (413 ± 532 *versus* 779 ± 834 ml, p = 0.009). All other intraoperative characteristics were similar between groups (urgency, CPB time, aortic clamp time, total operative duration, Table 1).

Postoperatively, drainage volumes were significantly reduced in the sealant group (985 ± 972 ml) *versus* the control group (1709 ± 1302 ml), p = 0.002 (Table 1), as was the duration of ICU stay and the total hospital stay (Table 1; both p = 0.03). In addition, a trend towards a reduced rethoracotomy rate was observed in the sealant group (1/48) *versus* the control group (6/54; p = 0.07). No adverse events related to the use of PEG sealant were reported during this study.

Overall, for the 102 procedures performed in this analysis, per patient cost-savings when adding a PEG sealant to the suture line (sealant group) *versus* no additive treatment (control group) for the anastomotic closure during aortic surgery were estimated at €1,943 (Table 2).

**Table 2:** **Estimated per-patient cost savingswhen using PEG sealantfor anastomotic closure duringaortic surgery**

**Surgical requirement**	**Unit cost**	**PEG sealant**	**Control (no sealant)**	**Cost saving using sealant**
**RBC**	€200/unit	3.6 units (€720)	5 units (€1,000)	€280
**FFP**	€160/unit	2.1 units (€336)	3 units (€480)	€144
**PEG sealant**	€237/2 ml application	2 ml (€237)	–	-€237
Rethoracotomy*	€2,000/procedure	0.02 (€41)	0.11 (€222)	€181
**ICU stay (days)**	€400/day	4.3	6.4	€840
**Hospital stay (days)**	€150/day	16.1	21	€735
**Overall estimated cost savingper patient**				**€1,943**

RBC, red blood cells (1 unit = 250 ml); FFP, fresh frozen plasma (1 unit = 200 ml); PEG, polyethylene glycol; ICU, intensive care unit.

*Operating room (OR)-associated costs only (i.e. anaesthesia, OR and staff time, but excluding extended intensive care or hospital stay).

Cost savings based on average reduced procedural requirements when using PEG sealant using typical costs at the Klinikum Oldenburg in the period January 2004 to June 2006.

## Discussion

The results of this single centre, retrospective case series demonstrate that adding a PEG sealant to the anastomotic closure of aortic Bentall procedures provides a beneficial effect on blood loss and transfusion requirements. Compared with sutures alone, PEG sealant significantly reduced postoperative drainage loss and transfusion requirements, with no additional adverse events. In addition, a trend towards a reduced rethoracotomy rate *versus* sutures alone was also observed. These benefits may have translated into substantial cost savings for aortic Bentall procedures over the 30-month study period (January 2004 to June 2006).

Several studies have recommended the use of surgical sealants for treating anastomotic suture lines in patients undergoing aortic reconstructions [2-5,8], and the positive findings reported here are supported by other retrospective analyses of sealant use in aortic reconstruction [9]. However, the case series reported here provides a more accurate overview of routine clinical practice than a controlled clinical study environment, and therefore the results are highly relevant to practising cardiac surgeons.

In addition to the benefits on clinical outcomes reported here, there are also important practical aspects that should be considered. Unlike haemostatic gelatins (with or without the use of thrombin), or haemostatic agents that induce platelet aggregation, the PEG sealant used in this study works independent of the clotting cascade, allowing its use in patients with severely inhibited coagulation. In addition, PEG sealant can and should be applied pre-emptively of any bleeding and requires the application of smaller volumes than other sealants, particularly if the spray applicator is utilised. This is advantageous as the use of smaller volumes of sealant can help to reduce the risk of stenosis, which is particularly important under the ostium of the left coronary artery. While the PEG sealant used provides a seal of great strength via covalent tissue bonds, it still retains flexibility. This allows normal physiological dilation without stiffening, thus avoiding any additional wall stress and weakening of the surrounding tissue [10]. The PEG sealant also begins to set in five seconds and is fully polymerised as a hydrogel in one minute [5], providing rapid sealing of the prosthesis. This prevents blood loss at unclamping, reducing the risk of further complications related to the extracorporeal circulation.

Cost calculations reported here are hypothesis generating, do not represent a detailed or formal analysis of cost effectiveness and warrant further confirmation in dedicated economic studies. Nevertheless, based on the estimated €1,943 per patient cost saving associated with use of PEG sealant in this analysis, it seems plausible that substantial reductions observed for some of the surgical requirements may have directly translated into procedural cost savings. The cost savings associated with the lower rate of transfusions conferred by the use of PEG sealant reported here add further to the data supporting the economic benefit of this intervention [11,12]. In particular, the suggested reduction in the rethoracotomy rate, which in itself influences the amount of RBC and FFP required as a result of the complication, results in cost savings from the use of PEG sealant far outweighing the cost of the treatment itself. In addition, the significant reductions in ICU stay and hospital stay associated with the use of PEG sealant would be expected to further reduce healthcare costs. We therefore consider the adjunctive use of the PEG sealant in this analysis of considerable economic benefit.

As with all clinical studies, there are limitations that should be considered. This was a retrospective analysis of non-blinded data, recorded at a single study centre. As such it may be open to potential bias. Three of the participating surgeons routinely added a PEG sealant to the anastomotic closure of aortic procedures, whereas the other three did not. Therefore, the results may have been influenced by particular surgeons’ experience and individual techniques. Furthermore, the fact that the data to be recorded for the efficacy and safety outcomes were not pre-defined, but taken out of the existing routine documentation, may also impact the suitability of the measured parameters. With regard to concomitant aspirin use, while it is likely that urgent or emergent cases did not have their aspirin discontinued before surgery and postoperative drainage volumes were increased as a result, the percentages of urgent/emergent cases were similar in the two groups (6/48 [12.5%] *versus* 8/54 [14.8%], respectively). Postoperative hypertension was not recorded during this study, so comparisons to establish the effectiveness of the PEG sealant in patients with postoperative hypertension were not possible. While the PEG sealant has been demonstrated to withstand supra-physiological pressures of up to 660 ± 150 mmHg [6], this is still an important clinical consideration that warrants further investigation in future studies.

## Conclusion

This retrospective analysis of aortic Bentall procedures in 102 patients over a 30-month period assessed the effect on blood loss and transfusion requirements of adding a PEG sealant to the anastomotic closure. The use of PEG sealant for suture-line reinforcement provided significant benefits on postoperative drainage and transfusion requirements *versus* sutures alone, with a trend towards a reduced rethoracotomy rate. These benefits may also translate into considerable cost savings. The clinically significant findings reported here warrant confirmation in prospective studies, which should also include the analysis of postoperative hypertension.

## Abbreviations

ICU: Intensive Care Unit; PEG: Polyethylene Glycol; RBC: Red Blood Cells; FFP: Fresh Frozen Plasma; CBP: Cardiopulmonary Bypass; CABG: Coronary Artery Bypass Graft; SD: standard deviation; EuroSCORE: European System for Cardiac Operative Risk Evaluation.

## Competing interests

EN has received personal compensation from Baxter Healthcare Corporation, the manufacturer of CoSeal®, for serving as a consultant and speaker. Baxter Healthcare Corporation financed the preparation and submission of this manuscript for publication. MS and OED declare that they have no competing interests.

## Authors’ contributions

EN and OED were responsible for the study concept, study design, performing surgical procedures, data analysis, and reviewing the manuscript. MS participated in the study concept and was responsible for data analysis, performing surgical procedures, and reviewing the manuscript. All authors read and approved the final manuscript.

## Supplementary Material

Additional file 1 **Video 1.** Intraoperative application of PEG sealant during an aortic surgical procedure using a spray applicator. Click here for file

Additional file 2 **Video 2.** Intraoperative application of PEG sealant during an aortic surgical procedure using a tip applicator. Click here for file
